# The sociodemographic, behavioral, reproductive, and health factors associated with fertility in Brazil

**DOI:** 10.1371/journal.pone.0171888

**Published:** 2017-02-10

**Authors:** Cesar Augusto Oviedo Tejada, Lívia Madeira Triaca, Flávia Katrein da Costa, Franciele Hellwig

**Affiliations:** 1 Posgraduate Program in Economics, Federal University of Pelotas, Pelotas, Rio Grande do Sul, Brazil; 2 Posgraduate Program in Epidemiology, Federal University of Pelotas, Pelotas, Rio Grande do Sul, Brazil; National Institute of Health, ITALY

## Abstract

High fertility rates among disadvantaged subgroups are a public health problem because fertility levels significantly affect socioeconomic conditions and a population’s welfare. This paper aims to analyze the sociodemographic, behavioral, and reproductive factors associated with fertility rates among Brazilian women aged between 15–49 years. A Poisson regression was used to analyze data from the 2006 PNDS (*Pesquisa Nacional de Demografia e Saúde da Criança e da Mulher*), which evaluates socioeconomic, demographic, geographic, reproductive, behavioral, and chronic disease variables. The results show that the following characteristics are positively associated with an increase in the number of children born: being aged 20–24, residing in the North, being nonwhite, not being in paid employment, having lower education levels, having lower socioeconomic status, being in a stable union, having the first sexual intercourse before the age of 16 and having the first child before the age of 20. Thus, it is important to implement efficient family planning policies targeting these subgroups in order to improve life conditions, reduce inequalities and avoid the adverse outcomes of high fertility.

## Introduction

High fertility rates among disadvantaged subgroups are a public health problem because fertility levels significantly affect socioeconomic conditions and a population’s welfare [1]. Fertility patterns strongly influence the probability of children’s survival, child morbidity and mortality, and maternal health [1,2].

The demographic transition is a phenomenon common to all countries and characterized by the reduction of the mortality rate followed by the reduction of the fertility rate, resulting in low population growth [3]. Brazil is crossing an intermediary stage of the demographic transition with low levels of mortality and decreasing fertility [4].

Brazil’s population recorded the highest rates of population growth between the years 1950 and 1970, approximately 3.0% per year. Later, there was a decrease in those rates, reaching 1.17% per year in the 2000s [5]. The total fertility rate decreased from 6.3 children per woman in 1960 to 1.9 child per woman in 2010 [6]. Replacement fertility is the number of births per woman that would result in stable population levels, which is 2.1 children per woman for most countries [3]. Currently, the fertility rate in Brazil is below the replacement level [7].

However, the Brazilian demographic transition occurs heterogeneously. Changes in fertility rates vary according to socioeconomic and demographic characteristics, with some subgroups increasing to high fertility levels and others decreasing to below-replacement fertility [7]. The *Pesquisa Nacional de Demografia e Saúde da Criança e da Mulher* (National Survey on Demographic and Health of Women and Children, also known as PNDS, 2006) [8] found that, on average, women from the North have 2.3 children per woman whereas the fertility rate was 1.7 children per woman in the South; women without educational achievement have more than 4 children per woman while those with more than 12 years of schooling have only 1 child per woman; according to skin color, the fertility rate was 1.98 children per nonwhite woman compared to 1.53 children per woman who self-declared as white.

Previous studies have analyzed the determinants of fertility in Brazil and found that fertility rates are lower for women from the urban population [5,9–12], with higher income [5,9,12], with access to technology (Internet and mobile phone) [9], who do not own their home [12], with higher education [5,9–12], who are white [5,9–12], who do not live in a stable union [5,13], who live in more developed regions [11], who are of the Christian religion [13], who attend church regularly [13], and who have paid employment [5,9,10]. Studies conducted in other countries show results very similar to those observed in Brazil [14,15].

Although there is an abundance of literature focusing on factors associated with fertility, the majority is composed of descriptive analysis with a focus on socioeconomic and demographic characteristics from a specific sample, such as women from an age group or a subnational region. Our analysis contributes to the literature by expanding the sample to all Brazilian women who are of reproductive age and includes variables of reproductive behavior.

The aim of the study was to contribute to the knowledge of socioeconomic, behavioral, and reproductive factors associated with fertility rates of Brazilian women using the data provided by the 2006 PNDS. Better understanding of this relationship allows for identification of proper governmental intervention and supports the formulation of public policies.

## Materials and methods

Data analyzed were from the 2006 PNDS [8], inserted in the 5th project phase MEASURE DHS (Demographic and Health Survey). The DHS is a global program that analyzes and provides data from low- and middle-income countries, focusing on the health and nutrition of women and children, and it is the most comprehensive Brazilian data source regarding sexual and reproductive health [16]. In the 2006 edition, 15,575 women of childbearing age between the ages of 15 and 49 were interviewed. The sample is representative of the five geographical regions of Brazil and of urban and rural areas. The fertility measure used was parity, the total number of children born alive as of the interview date. The independent variables were divided into six groups: (1) geographic, (2) demographic, (3) socioeconomic, (4) reproductive, (5) health, and (6) behavioral.

The first group of independent variables contains information from the region where the participants live (North, Northeast, Southeast, South, and Midwest) and the area of their residence (urban or rural). The second group includes race (white and nonwhite), age (15–19, 20–24, 25–29, 30–34, 35–39, 40–44 and, 45–49 years old), marital status (cohabitating or not), and migration (0–5, 6–10, and over 10 years), which refers to the time period of residence in the city.

The third group of variables includes education (less than 1 year, 1–4, 5–8, 9–11, and 12 or more), socioeconomic status (in quintiles), and employment (yes or no). The socioeconomic level used corresponds to the DHS Wealth Index, which is the wealth index developed for the DHS project’s research.

The fourth group, reproductive variables, contains the most important differentials of the DHS surveys for the fertility analysis. The variables analyzed were age of first sexual intercourse (less than 15 years old; 15 to 17 years old; and equal to or more than 18 years old), the age of the first birth (less than 20 years old; 24–29; 30–39; and 40–49 years old) and using contraception at first sexual intercourse (yes or no).

Regarding health measures, a variable was included addressing the diagnosis of chronic diseases (none and one or more). The following diseases were studied: hypertension or heart disease, diabetes, depression, anxiety or insomnia, bronchitis or asthma, arthritis or rheumatism, and anemia. Finally, behavioral variables included in the model were religion (none, Catholic, Evangelical, and others) and information, whether participants have seen, heard, or read something about the prevention of pregnancy in any media outlet (yes or no).

The data were analyzed using Stata software version 12.0 [17]. The statistical analysis consisted of the description of the sample and included mean parity and high parity (%) for all variables used in the study. Mean parity is the number of children born alive divided by the number of women sampled and high parity is the percentage of women who have parity ≥ 5. The relationship between the fertility rate and sociodemographic, behavioral, and reproductive health was assessed through crude and adjusted analyses. Both analyses were performed using Poisson regression, considering different observation exposure times.

## Results

Table 1 describes the composition of the sample, the mean parity and the high parity for each category of the variables analyzed. The sample was divided proportionally among the five geographical regions of the country, with 71% of individuals residing in urban areas. Most participants (61.2%) do not consider themselves white and self-declared as black, brown, yellow, or indigenous. Regarding age, the sample was divided proportionally with frequencies ranging from 11.3% (45–49 years) to 16.1% (20–24 years). A larger portion is in a stable union (64.2%), identify as Catholic (81.7%), and have resided in the city for over 10 years (70.9%). More than one third of the sample studied from 9 to 11 years (35.3%), while only 3.5% studied less than 1 year. Approximately 53.4% of women said they did not have another job in addition to household chores.

**Table 1:** Sample description and average fertility rate according to geographic, demographic, socioeconomic, reproductive, behavioral, and health variables.

Variables	Sample composition		Mean parity	High parity %
	N	%		
**Total**	15,534	100.00	1.77	6.79
**Region (n = 15,534)**				
North	2,587	16.65	2.15	12.14
Northeast	3,156	20.32	1.82	8.87
Southeast	3,334	21.46	1.63	4.98
South	3,302	21.26	1.60	3.85
Midwest	3,155	20.31	1.73	5.32
**Geographic area (n = 15,534)**				
Rural	4,500	28.97	2.16	10.89
Urban	11,034	71.03	1.61	5.12
**Race (n = 15,392)**				
Nonwhite	9,416	61.17	1.90	8.89
White	5,976	38.83	1.55	3.43
**Age (n = 15,534)**				
15–19	2,487	16.01	0.18	0.00
20–24	2,500	16.09	0.83	0.48
25–29	2,431	15.65	1.57	2.71
30–34	2,299	14.80	2.14	7.31
35–39	2,092	13.47	2.50	10.13
40–44	1,968	12.67	2.77	13.06
45–49	1,757	11.31	3.14	19.35
**Marital status (n = 15,533)**				
Single	5,568	35.85	0.85	3.21
Cohabitating	9,965	64.15	2.28	8.79
**Migration (n = 15,484)**				
0–5 years	2,739	17.69	1.59	5.26
6–10 years	1,768	11.42	1.86	6.84
More than 10 years	10,977	70.89	1.79	7.12
**Years of schooling (n = 15,514)**				
Less than 1 year	537	03.48	4.04	32.59
1–4 years	3,189	20.69	2.96	17.06
5–8 years	4,667	30.28	1.76	5.21
9–11 years	5,449	35.35	1.08	1.45
12 years or more	1,572	10.20	0.99	0.32
**Wealth Index (n = 14,343)**				
1° quintile (lowest)	2,592	18.07	2.29	13.43
2°	2,772	19.33	1.96	8.01
3°	2,897	20.20	1.68	4.63
4°	2,994	20.87	1.45	2.57
5° quintile (highest)	3,088	21.53	1.15	0.84
**Employment (n = 15,531)**				
No	8,287	53.36	1.79	7.59
Yes	7,244	46.64	1.74	5.88
**First sexual intercourse (n = 13,597)**				
< 15 years old	1,967	14.47	2.58	14.59
15–17 years old	5,711	42.00	2.05	8.54
≥18 years old	5,919	43.53	1.72	4.17
**First birth (n = 11,052)**				
Less than 20 years old	6,210	56.19	2.84	13.67
20–29 years old	4,415	39.95	2.06	4.44
30–39 years old	423	03.83	1.32	0.00
40–49 years old	4	00.04	0.50	0.00
**Contraception at first intercourse(n = 9,384)**				
No	6,464	68,88	2.69	13.85
Yes	2,920	31,12	1.87	3.18
**Chronic diseases (n = 15,534)**				
None	6,939	44,67	1.49	5.13
One or more	8,595	55,33	2.00	8.13
**Religion (n = 15,513)**				
None	285	01.84	1.53	5.26
Catholic	12,678	81.73	1.84	7.32
Evangelical	2,235	14.41	1.46	4.56
Others	315	02.03	1.23	2.54
**Information(n = 15,534)**				
No	2,328	14.99	2.58	15.55
Yes	13,206	85.01	1.63	5.25

Source: PNDS, 2006, Brazil. Authors elaboration.

Regarding reproductive variables, 42% of women had sexual intercourse for the first time between the ages of 15 and 17, 56.2% of them had become pregnant for the first time before the age of 20 and approximately 70% did not use any contraception at first sexual intercourse. Among the women surveyed, 55.3% said they have been diagnosed with one or more chronic disease, and 85.0% said they received information about preventing pregnancy from a media outlet. (Table 1)

There is a higher mean parity among women from the North (2.15 children per woman), living in rural areas (2.16 children per woman), in a stable union (2.28 children), nonwhite (1.9 children per woman), with less than one year of schooling (4.04 children per woman), in a lower socioeconomic level (2.29 children per woman) and who had one or more chronic disease (2 children per woman). Parity increases with the age; women between the ages of 15 and 19 have on average 0.18 children per woman while women at the end of their reproductive period (between the ages of 45 and 49) have a mean parity of 3.14. According to reproductive variables, higher parity was found for women who had become pregnant for the first time before age 20 (2.84 children per woman), who did not use contraception at first sexual intercourse (2.69 children per woman) and had first sexual intercourse before age 15 (2.58 children per woman). (Table 1).

The percentage of high parity is higher for the most vulnerable groups. High parity is more frequent among women with less than one year of schooling (32.6%), who are from the lowest socioeconomic level (13.4%), and who live in the North (12.1%) and in rural areas (10.9%). In relation to reproductive behavior, high parity levels are more frequent for women who did not use contraception at their first sexual intercourse (13.9%), who had the first sexual intercourse before age 15 (14.6%), and who had the first child before the age of 20 (13.7%). (Table 1).

Table 2 presents the results of crude and adjusted analyses. The analyses were prepared using estimates of Poisson regression, and the incidence rate ratios (IRR) of the number of children are presented.

**Table 2:** Crude and adjusted incidence rate ratios (IRRs) estimated using Poisson regression.

	Crude Analysis			Adjusted Analysis		
	IRR	95% CI	P	IRR	95% CI	P
**Age**			<0.001			<0,001
15–19	1			1		
20–24	2.50	(2.05–3.05)		1.09	(0.96–1.23)	
25–29	3.10	(2.56–3.75)		1.07	(0.95–1.20)	
30–34	3.45	(2.87–4.15)		1.07	(0.95–1.20)	
35–39	3.24	(2.68–3.91)		1.01	(0.89–1.14)	
40–44	3.18	(2.66–3.81)		0.96	(0.84–1.08)	
45–49	3.08	(2.55–3.72)		0.92	(0.81–1.04)	
**Region**			<0.001			<0,001
North	1			1		
Northeast	0.82	(0.75–0.89)		0.94	(0.89–0.99)	
Southeast	0.62	(0.57–0.68)		0.94	(0.87–1.00)	
South	0.63	(0.59–0.68)		0.88	(0.83–0.93)	
Midwest	0.75	(0.70–0.80)		0.90	(0.85–0.94)	
**Employment**			<0.001			<0,012
No	1			1		
Yes	0.82	(0.78–0.86)		0.95	(0.92–0.99)	
**Years of schooling**			<0.001			<0,001
Less than 1 year	1			1		
1–4 years	0.83	(0.75–0.92)		0.95	(0.88–1.03)	
5–8 years	0.66	(0.59–0.72)		0.88	(0.81–0.95)	
9–11 years	0.43	(0.38–0.47)		0.79	(0.73–0.86)	
12 years or more	0.29	(0.25–0.32)		0.79	(0.72–0.87)	
**Race**			<0.001			0,010
Nonwhite	1			1		
White	0.79	(0.75–0.83)		0.94	(0.91–0.98)	
**First sexual intercourse**			<0.001			<0,001
< 15 years old	1			1		
15–17 years old	0.71	(0.67–0.75)		0.86	(0.81–0.91)	
≥18 years old	0.47	(0.44–0.50)		0.80	(0.75–0.85)	
**First birth**			<0.001			<0.001
15–19 years old	1			1		
20–29 years old	0.61	(0.58–0.63)		0.75	(0.72–0.78)	
30–39 years old	0.36	(0.32–0.39)		0.47	(0.42–0.52)	
40–49 years old	0.09	(0.02–0.39)		0.02	(0.00–0.23)	
**Contraception at first intercourse**			<0.001			0.022
No	1			1		
Yes	0.72	(0.68–0.76)		0.95	(0.92–0.99)	
**Marital status**			<0.001			<0,001
Single	1			1		
Cohabitating	2.09	(1.92–2.28)		1.11	(1.04–1.17)	
**Wealth Index**			<0.001			<0,001
1°	1			1		
2°	0.84	(0.79–0.90)		0.89	(0.84–0.94)	
3°	0.72	(0.67–0.77)		0.84	(0.79–0.90)	
4°	0.61	(0.56–0.66)		0.77	(0.73–0.82)	
5°	0.46	(0.42–0.50)		0.74	(0.69–0.80)	

Source: PNDS, 2006. Brazil. Own elaboration.

Note: Confidence interval of 95% and chi-square tests for heterogeneity and linear trend for ordinal variables.

In the crude analysis, all variables were statistically significant in relation to fertility. In the adjusted analysis, no statistically significant differences were observed in fertility in the following variables: religion, geographic area, chronic disease, information, and migration.

Parity tends to increase at the beginning of adulthood; it is 9% higher among women aged 20–24 years, compared to women aged 15–19 years. After the age of 24, parity decreases as women age. The North region has higher parity, while the Northeast and Southeast regions have 6% fewer children in comparison. Women from the South and Midwest have, respectively, 12% and 10% fewer children compared to the North.

There has been a downward trend regarding the number of children of women with higher education. Women with 12 or more years of schooling have a 21% lower IRR compared to those who have less than one year of schooling. The later a woman has sexual intercourse for the first time or becomes pregnant for the first time, the lower the number of children. Among those who had sexual intercourse for the first time at more than or equal to 18 years old, the IRR is 20% less than for those who were sexually active before age 15. Women who had their first child between 30 and 39 years old have 53% fewer children, compared to those who had their first child between 15 and 19 years. Women who had their first child between the ages of 40 and 49, have IRR approximately 98% lower than those in the younger age groups. Use of contraception at first sexual intercourse also shows a negative impact. Women who did not use any contraception have higher IRRs.

It is possible to observe a decreasing trend in the number of children with increasing socioeconomic status. Among the richest 20%, the incidence rate is approximately 26% lower than among the poorest 20%.

## Discussion

There is a relationship between development and fertility. Regions that declined in fertility with a high level of development showed an extraordinarily fast decline in fertility rates [18] and have completed the process of demographic transition, while in less developed regions, this process is still in progress [4]. In Brazil, as in many other countries, fertility rates have decreased over the past decades. However, this decrease occurred in different proportions among Brazilian women. The fertility profile is influenced mainly by education, socioeconomic status, age, macro-region, marital status, age of first sexual intercourse, and age at first birth.

Brazil has a rejuvenated pattern of fertility; women start having children early and soon reach desired fertility [6]. This study indicates that the fertility peak occurs between the ages of 20 and 24, 9% higher than for women 15–19 years old. After the age of 24, fertility decreases as women age. Similar results were found in other studies of Brazil [10,13] and other Latin America countries [19,20].

The differences in sexual and reproductive patterns among Brazilian regions reflect the differences in socioeconomic status, culture, and access to health services and contraceptive methods [21]. Compared with women living in the North, women from all other regions have lower fertility levels. The lowest level among Brazilian regions was found in women living in the South. Other studies have found evidence of a lower level of fertility for women living in the Southeast region [5,12]. One study found a lower level of fertility for women from the South region after 2004 [11].

Schooling has a great impact on fertility in low- and middle-income countries. Higher levels of education help women to make more informed decisions [22]. Moreover, it is possible that education improves literacy and increases the effectiveness of family planning methods and contraception [14,15]. Data from 2006 PNDS show that women with higher education have a 20% lower IRR than women with less education (less than 1 year). Several studies of the factors associated with fertility of Brazilian women found similar results [5,11,12], indicating that each level of education significantly reduces the number children. Similar results were also found in the United States [23], Singapore [24], Ethiopia [25], Ecuador [26], Peru [15,27], Colombia [26], and other countries in Latin America [19] and the world [14,28]. However, this is not observed in European countries, where the increase in education levels causes women to increase their investments in the human capital of their children and does not necessarily cause a decrease in desired fertility [29]. However, a study on fertility of women from Portugal found a negative relationship between education and fertility regarding higher education levels [30].

Since the age of first sexual experience and first birth are linked to duration of exposure to risk of pregnancy, these factors are directly associated with the number of children born. It is noteworthy that women who had their first sexual intercourse or their first pregnancy at older ages have fewer children. Similar results were found in other studies in other countries [25].

The relationship between socioeconomic status and fertility is complex because it depends on other factors such as access to public services and the preferences of parents about their own consumption and their children’s consumption. Therefore, families from different socioeconomic groups respond differently to increasing incomes [31]. Several studies have identified socioeconomic status as an important factor associated with fertility; a family would restrict fertility to increase investments per child. Evidence of this inverse relationship between socioeconomic status and number of children was found in Brazil [5,9–12], the United States [23], Singapore [24], Ecuador [26], Colombia, and Peru [15]. Some other studies have found that income has a positive effect on fertility [1,9,32], but it is important to consider the indirect effects of income through other determinants such as mortality and education [1].

Another important factor associated with fertility is marital status. Women who are cohabitating have more children than those who are not. Similarly, age of first sexual intercourse, age at first birth, and age at marriage increase the risk of pregnancy [1]. Our results are consistent with earlier studies in Brazil [5,12], Colombia, and Peru [15].

Despite the existence of an extensive literature on the subject, our study contributes to the literature by using the most complete database available to study female fertility in Brazil, the 2006 PNDS allows us to include in the analysis, besides socioeconomic and demographic variables, variables related to female reproductive behavior, through a nationally representative sample.

Our results highlight the relevance of including reproductive variables and show that some variables that were historically significant such as religion [13], migration [33] and area of residence [5,12] lost its importance in the analysis of fertility.

Brazil still has inequalities in fertility patterns among population groups. Studying the factors associated with fertility is essential to the development of public policies aiming to reduce this inequality. The government should promote programs that seek to reduce the fertility of the groups in which it is still high. For this, access to education and family planning services are essential.

In addition, the decline in fertility has reduced dependency ratios of children and adolescents in Brazil, providing the so-called “demographic window of opportunity” that will close when the increase in dependency ratios of older people, caused by the decrease in mortality, overcomes the benefit of reduction in fertility. The country should invest in policies regarding education, health, and employment so the gains from the demographic dividend will become permanent [4].

The results above confirm the importance of the PNDS for the study of the reproductive behavior of Brazilian women. However, it is important to consider that analyzing the factors associated with fertility using cross-sectional data is not a complete representation of the reproductive cycle. Longitudinal data allows greater stability in the indicators, as observations would extend through women's life cycles [34]. Moreover, it is important to consider the presence of women who have not completed their reproductive cycle in the sample. The analysis of a sample of women who have completed their reproductive cycle (approximately 45 years) would generate more accurate estimates of the total number of children [5].Given the limitations on the structure of the cross-sectional data, there remains a need for further studies that address women’s entire reproductive period. The use of longitudinal data would be an advance in the analysis of the determinants of fertility.
